# Immunopathogenesis of Immune-Mediated Necrotizing Myopathy: Insights Drawn from Pathological Phenotypes and Mechanistic Elaboration

**DOI:** 10.3390/biomedicines14092087

**Published:** 2026-09-16

**Authors:** Xiaoke Wu, Junjie Qian, Xin Wang, Na Ji, Yingning Li, Shuying Chen, Qingjie Li, Meirong Liu, Qi Fang

**Affiliations:** 1 Department of Neurology, The First Affiliated Hospital of Soochow University, Suzhou 215006, China; yoyowuxiaoke@163.com (X.W.); 18762913755@163.com (J.Q.); wangxin19902411@163.com (X.W.); regina5279@163.com (N.J.); chenshuying2024@163.com (S.C.); 2 Suzhou Medical College of Soochow University, Soochow University, Suzhou 215123, China; 2230506073@stu.suda.edu.cn; 3 Department of Immunology, The Affiliated Hospital of Xuzhou Medical University, Xuzhou 221000, China; liqingjiegxy@126.com

**Keywords:** Immune-mediated necrotizing myopathy, anti-SRP autoantibodies, anti-HMGCR autoantibodies, humoral immunity, innate immunity, necroptosis, targeted therapy

## Abstract

Immune-mediated necrotizing myopathy (IMNM) is a distinct subtype of idiopathic inflammatory myopathies (IIMs), classified by serum autoantibody status into anti-signal recognition particle (anti-SRP)-positive, anti-3-hydroxy-3-methylglutaryl-coenzyme A reductase (anti-HMGCR)-positive, and seronegative subgroups. Its hallmark is a pathological paradox: extensive myofiber necrosis and regeneration accompanied by strikingly sparse inflammatory infiltrates, a feature that distinguishes IMNM from dermatomyositis and inclusion body myositis. In this review, we integrate preclinical and clinical evidence to build an immune-mechanistic pathogenesis model grounded in pathological observations: anti-SRP and anti-HMGCR autoantibodies trigger myofiber injury via both complement-dependent and complement-independent pathways; necrotic myofibers release damage-associated molecular patterns (DAMPs) that activate Toll-like receptor signaling, thereby establishing a complement-independent, self-perpetuating injury-amplification loop; T-cell-dominated adaptive-immune responses, characterized by Th1-M1 pathway activation alongside widespread T-cell exhaustion, may only function as a secondary modulator; and aberrant vascular remodeling coupled with impaired tissue regeneration contributes to long-term prognosis. Variations in macrophage polarization, angiogenic potential, and malignancy risk between anti-SRP-positive and anti-HMGCR-positive IMNM may constitute intrinsic drivers underlying their disparate histopathological features, clinical trajectories, and treatment responses. This model accounts for the limited therapeutic benefit observed with complement-blockade monotherapy and proposes that regimens combining autoantibody suppression with the inhibition of necrosis-driven injury amplification could yield improved clinical outcomes, providing a theoretical basis for pathological subtyping and stratified therapy in IMNM.

## 1. Introduction

### 1.1. Epidemiology and Subtype Distribution

IMNM, also known as necrotizing autoimmune myopathy (NAM), is a distinct clinicopathological subtype of IIM [1]. The European Neuromuscular Centre (ENMC) formally defined IMNM as an independent disease entity in 2003 [2]. According to the serum autoantibody profile, IMNM can be categorized into three subgroups: anti-SRP autoantibody-positive type, anti-HMGCR autoantibody-positive type, and seronegative type [3,4,5]. As a major subtype of IIM, IMNM accounts for 20–38% of all IIM cases. The overall incidence of IIM ranges from 9 to 14 per 100,000 individuals, with heterogeneous subtype distribution: anti-SRP-positive cases account for 18–39%, anti-HMGCR-positive cases for 24–45%, and seronegative cases for approximately 20% [6,7].

IMNM predominantly affects adults aged 40 to 50 years. The anti-SRP subtype exhibits a female predominance, with a male-to-female ratio ranging from 1:1.6 to 1:3.6, while the anti-HMGCR subtype presents a bimodal age distribution.5 Geographic and ethnic disparities significantly influence disease patterns. HMGCR-positive IMNM in European and American patients is strongly associated with statin exposure, whereas a larger proportion of Asian patients develop non-statin-related IMNM. Moreover, patients with the anti-SRP subtype generally have more severe disease and carry a higher risk of interstitial lung disease [8].

### 1.2. Clinical Manifestations

IMNM is characterized by acute or progressive symmetric proximal limb weakness with myogenic changes on electromyography. The anti-SRP subtype presents the most severe phenotype, manifesting as acute to subacute weakness that frequently involves cervical, respiratory, bulbar, and facial muscles, accompanied by severe muscular atrophy and markedly elevated creatine kinase (CK). In contrast, the anti-HMGCR subtype progresses more slowly; nevertheless, at least 50% of patients fail to achieve full functional recovery, and refractory disease with persistently elevated CK is common [7]. Myalgia is the predominant clinical symptom in Chinese patients with seronegative IMNM [9].

### 1.3. Clinical Challenges

The clinical diagnosis and management of IMNM face substantial challenges. Muscle injury frequently results in severe disability, and treatment responses show marked heterogeneity: approximately 50% of patients respond favorably to conventional immunosuppressive therapy, whereas younger individuals develop more refractory disease with slower recovery. The anti-SRP subtype requires more intensive immunosuppression compared with the anti-HMGCR subtype, and patients with seronegative IMNM may follow distinct treatment trajectories [10]. A major barrier to evidence-based treatment is the extreme scarcity of randomized controlled trials (RCTs). To date, only one RCT, the phase II trial of the C5 inhibitor zilucoplan, has been completed. However, this trial failed to meet its primary endpoint, indicating that complement blockade alone is insufficient to control the disease. The negative outcomes of complement inhibitor trials reflect an incomplete understanding of the underlying pathophysiology [11]. These dilemmas highlight the urgency of carefully examining IMNM from the perspective of immune mechanisms.

Existing IMNM reviews primarily focus on clinical features and treatments or separately illustrate the pathogenic mechanisms of anti-SRP and anti-HMGCR antibodies. Several studies have addressed individual pathways including complement activation, macrophage responses, and programmed cell death. However, few systematic reviews have integrated multi-layered immune crosstalk to explain the typical IMNM pathological phenotype characterized by extensive myofiber necrosis with mild inflammatory infiltration. In this review, we summarize recent progress in humoral, innate, and adaptive-immune mechanisms underlying IMNM, aiming to provide a reference for further mechanistic investigations.

### 1.4. Literature Search Strategy

For this narrative review, we retrieved relevant studies published between 2013 and 2026 from PubMed, Embase, and Web of Science. Key search terms included IMNM, anti-SRP antibody, anti-HMGCR antibody, humoral immunity, innate immunity, cellular immunity, and targeted therapy. Original experimental studies, clinical trials, and authoritative reviews were included, whereas letters and case reports lacking sufficient mechanistic evidence were excluded. As a narrative review, our synthesis reflects the authors’ selection and interpretation of the available evidence rather than a systematic search with quantitative synthesis; consequently, the possibility of selection bias cannot be fully excluded.

## 2. IMNM: A Distinct Immunopathological Entity

### 2.1. Pathological Hallmarks

The core pathological features of IMNM consist of extensive myofiber necrosis and regeneration with only sparse infiltration of CD68-positive macrophages, accompanied by an absence of the typical invasion of non-necrotic myofibers by CD8-positive T cells. In addition, deposition of the complement membrane attack complex C5b-9 (MAC) on the sarcolemma is frequently observed, and major histocompatibility complex class I (MHC-I) molecules are predominantly expressed in a focal and cytoplasmic pattern [12,13].

Notably, prominent pathological heterogeneity exists across different subtypes. The anti-SRP-positive subtype generally presents more severe necrosis and regeneration, while sarcolemmal C5b-9 deposition is less common. By contrast, perivascular inflammatory infiltration and aggregated MHC-I expression are more frequently detected in the anti-HMGCR-positive subtype. Recent studies have further revealed that patients positive for anti-SRP or anti-HMGCR antibodies who fail to meet the EULAR/ACR classification criteria exhibit milder necrosis and regeneration, along with markedly fewer p62-positive myofibers encoded by SQSTM1. These patients still retain subtype-specific pathological manifestations: MAC expression on the sarcolemma is rarely seen in anti-SRP-positive cases; anti-HMGCR-positive cases show low MHC-I levels and limited MAC deposition [14,15,16].

### 2.2. Immunological Differences from Other Inflammatory Myopathies

The pathological features described above can be precisely validated at the molecular level by bulk transcriptomic profiling of muscle tissues. Distinct clinical and autoantibody-defined subtypes of myositis exhibit characteristic expression profiles of cytokines, cytokine receptors, and immune checkpoint genes, as demonstrated by a large-scale transcriptomic analysis covering 338 immune-related genes across multiple IIM subgroups [17]. These subtype-specific molecular signatures reflect divergent inflammatory patterns among dermatomyositis, inclusion body myositis, anti-synthetase syndrome, and IMNM, and can serve as specific biomarkers for the molecular subtyping of myositis.

Dermatomyositis (DM) is an interferon-driven disease. Compared with healthy controls, tissue interferon-stimulated gene 15 (ISG15) expression in patients is upregulated by 101-fold. Pathologically, it is characterized by perifascicular MHC-I expression and capillary-targeted MAC deposition. Additionally, peripheral monocytic sialic acid binding Ig-like lectin 1 (SIGLEC1) expression shows a significant positive correlation with disease activity [17].

Inclusion body myositis (IBM) is predominantly driven by T-cell immune responses, with markedly elevated C-X-C motif chemokine ligand 10 (CXCL10) expression in lesions. CD8^+^ T cells actively invade non-necrotic myofibers, and circumferential upregulation of MHC-I is observed on the surface of all myofibers. The molecular and pathological phenotypes of anti-synthetase syndrome (ASS) occupy an intermediate position between DM and IBM, featuring perifascicular necrosis, moderate type I interferon activation, and unique transcriptomic signatures [17].

In sharp contrast to these subtypes, IMNM displays highly distinct molecular signatures. Patients with IMNM show minimal type I interferon activation, with ISG15 expression downregulated approximately 12-fold relative to control subjects. Meanwhile, peripheral monocytic SIGLEC1 is expressed at low levels, showing no obvious difference from healthy individuals (1246 vs. 1200 mAb/cell). Transcriptomic analyses confirm that the gene expression profile of IMNM is not dominated by activated inflammatory pathways; instead, it is characterized by a high expression of genes associated with tissue repair and debris clearance, with SPP1 as the core molecule. Further pathway enrichment analyses demonstrate no prominent activation of canonical inflammatory pathways related to T helper 1, T helper 2, or T helper 17 cells. The highly expressed secreted phosphoprotein 1 gene (SPP1) correlates primarily with markers of muscle regeneration rather than lymphocytic infiltration, which also accounts for the low activation of type I interferon-related biomarkers [17,18,19]. Mechanistic inferences drawn from these molecular signatures place IMNM in a unique position within the IIM spectrum. Unlike IFN-driven DM and T-cell-mediated injury in IBM, IMNM follows a distinct damage cascade initiated by antibody-dependent necrosis and subsequently amplified by innate immune responses, with a more limited, though not absent, contribution of adaptive immunity.

## 3. Humoral Immunity: The Initiating Role of Autoantibodies

### 3.1. CXCL13 and In Situ B Cell Responses

C-X-C motif chemokine ligand 13 (CXCL13) is a potent B-cell chemokine that is upregulated in the muscle tissue of anti-SRP-positive IMNM patients. Nevertheless, intramuscular infiltration of B cells and plasma cells remains sparse and can only be detected in a subset of muscle biopsies, which is in marked contrast to other autoimmune myopathies. The local humoral response in IMNM is predominantly driven by autoantibodies generated in secondary lymphoid organs; skeletal muscle primarily acts as the target of immune attack rather than a site for sustained local antibody production. This finding has therapeutic implications, supporting systemic B-cell-targeted strategies while casting doubt on approaches that solely target the intramuscular immune microenvironment [20].

### 3.2. Complement-Dependent Myofiber Necrosis

Anti-SRP and anti-HMGCR autoantibodies trigger muscle injury via two pathways: cell-surface binding and intracellular internalization. Anti-SRP antibodies exert limited effects alone; in the presence of complement, they bind myofiber antigens, activate the classical complement pathway, deposit MAC on the sarcolemma, and ultimately induce myofiber lysis and necrosis [21]. Landmark passive transfer experiments have firmly established this complement-dependent pathway: the injection of patient-derived IgG into mice recapitulates muscle weakness, whereas disease severity is attenuated in C3-deficient mice. Widespread complement deposition also damages capillaries and triggers local ischemia, underscoring the central role of humoral immunity [22].

### 3.3. Autoantibody Internalization

Both anti-SRP and anti-HMGCR antibodies can penetrate myofibers and disrupt intracellular processes. Anti-HMGCR antibodies interfere with cholesterol metabolism, while anti-SRP antibodies block protein transport [23,24,25]. The antibodies also directly induce the atrophy of mature myofibers by upregulating MAFbx (ATROGIN1) and TRIM63 (MURF1), accelerating sarcomeric protein degradation. Meanwhile, they stimulate myotubes to secrete interleukin-6 (IL-6) and tumor necrosis factor alpha (TNF-α), elevate levels of reactive oxygen species (ROS), including mitochondrial superoxide, suppress the secretion of IL-4 and IL-13 by myoblasts, block myoblast fusion, and impair muscle regeneration. These findings challenge the traditional view that autoantibodies act exclusively extracellularly through complement activation [26].

### 3.4. Targeted Therapies for the Humoral Immunity Axis

**Figure 1:**
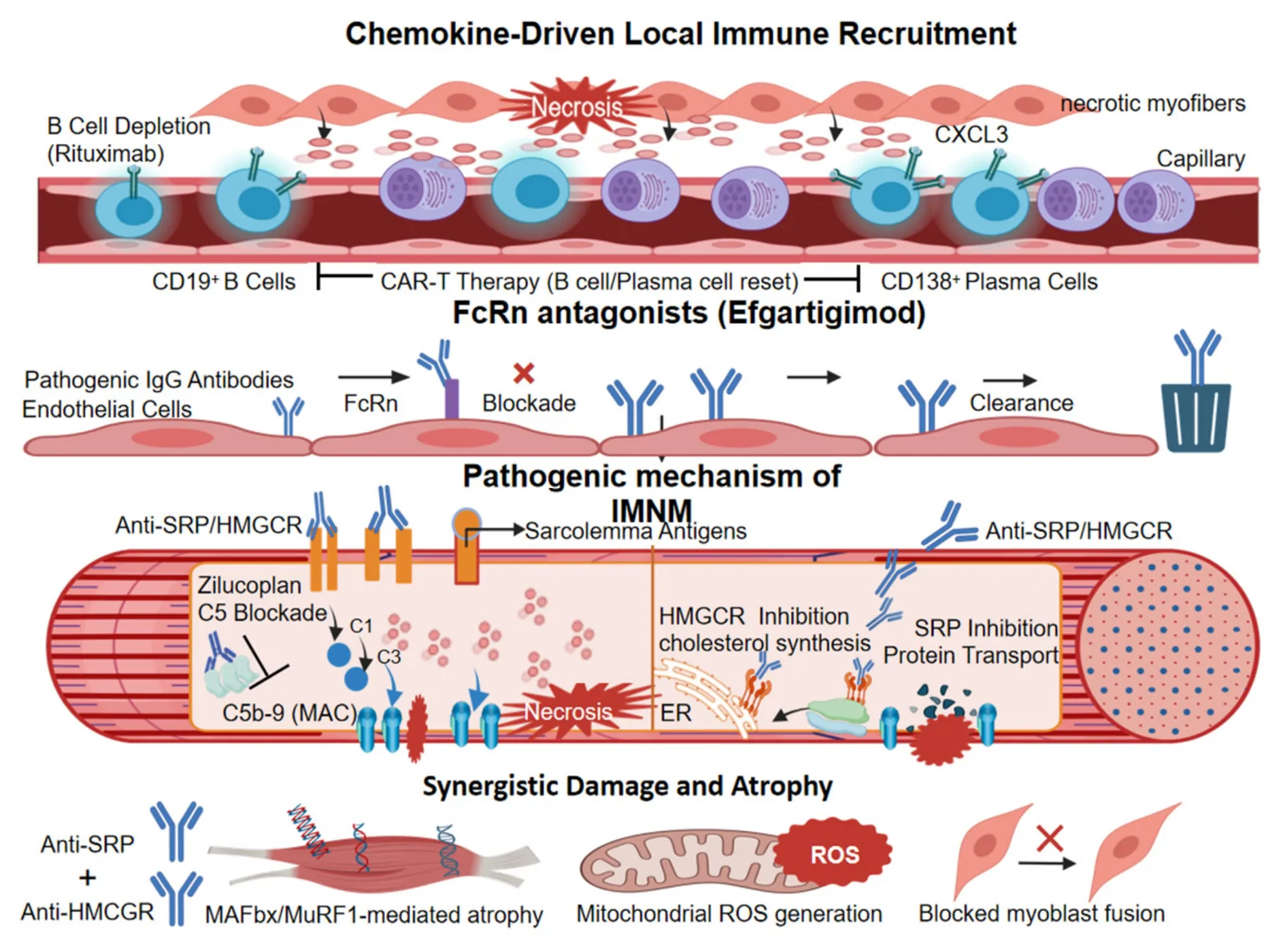
Pathogenic mechanisms and targeted therapeutic interventions in immune-mediated necrotizing myopathy (IMNM). Upper panel: CXCL13-driven recruitment of CD19^+^ B cells and CD138^+^ plasma cells within muscle tissue, where pathogenic autoantibodies are produced; therapeutic strategies including rituximab-mediated B-cell depletion and B-cell-directed CAR-T therapy are indicated. Middle panel: FcRn antagonists (efgartigimod) accelerate the clearance of circulating pathogenic IgG autoantibodies by blocking FcRn-dependent antibody recycling. Lower panel: Anti-SRP and anti-HMGCR autoantibodies target sarcolemmal antigens to trigger complement-driven myofiber necrosis via MAC (C5b-9) formation, which can be suppressed by the C5 inhibitor zilucoplan. Intracellular disruption of SRP-dependent protein transport or HMGCR-mediated cholesterol synthesis further aggravates myocyte injury. Combined autoantibody-induced insults induce synergistic muscle damage, including MAFbx/MuRF1-dependent muscle atrophy, mitochondrial ROS overproduction, and impaired myoblast fusion.

With the elucidation of humoral immune mechanisms, treatment strategies have evolved from broad-spectrum immunosuppression to targeted intervention. Efgartigimod antagonizes neonatal Fc receptor (FcRn) to degrade pathogenic IgG [27]. Rituximab depletes CD20^+^ B cells (a systematic review of 34 cases reported an overall response rate of 61.8%) [28]. Chimeric antigen receptor T cell (CAR-T) therapy is emerging as a promising approach: CD19-targeted CAR-T eliminates peripheral B cells and restores physiological B-cell homeostasis, while B-cell maturation antigen (BCMA)-targeted CAR-T has achieved sustained remission in refractory anti-SRP-positive patients (in one reported case, CK levels dropped from 4778 to 260 IU/L, and anti-SRP antibodies turned negative during an 18-month follow-up period) [29,30]. These therapies show promising potential, yet large-scale randomized controlled trials (RCTs) are required for validation. (The humoral immune mechanisms are illustrated in Figure 1).

## 4. Innate Immunity: The Necrosis Amplification Loop

### 4.1. Complement: The Initiator of Necrosis

MAC deposition directly triggers myofiber lysis. As a potent chemokine, the cleavage product C5a recruits CD68-positive macrophages in concert with danger signals from necrotic lesions. Macrophages with high C1QC expression are highly enriched in IMNM lesions; their phagocytic and antigen-presenting functions are detailed in Section 4.4. Complement activation serves as an early critical event linking humoral initiation and innate immune amplification [31].

### 4.2. DAMPs: Self-Amplifying Inflammatory Engine

Necrotic myofibers release DAMPs, which are proposed to act as a central driver of the inflammatory cascade in IMNM. In an observational single-center cohort study from Australia based on the South Australian Myositis Database (SAMD), skeletal muscle frozen biopsies and peripheral serum samples were used; sarcoplasmic high mobility group box 1 (HMGB1) in muscle was semi-quantified by immunohistochemistry, whereas circulating HMGB1 in serum was measured by enzyme-linked immunosorbent assay (ELISA). This study demonstrated elevated HMGB1 in both muscle tissue and serum among IMNM patients, and sarcoplasmic HMGB1 from muscle biopsies was positively correlated with the severity of muscle necrosis (rs = 0.62–0.77; *p* < 0.01) and macrophage infiltration (rs = 0.63; *p* < 0.001), and negatively correlated with MMT-8-tested muscle strength (MMT-8; rs = −0.57; *p* = 0.03). Limitations include its single-center design without external validation, small sample size (n = 14) for strength-related correlation analysis, and non-synchronized sampling time between muscle biopsy and serum collection (no correlation found between sarcoplasmic and serum HMGB1) [32]. In a 2025 Chinese single-center cohort, single-cell RNA sequencing (scRNA-seq) and bulk RNAseq combined with immunofluorescence revealed marked local upregulation of the macrophage migration inhibitory factor (MIF)-cluster of differentiation 74 (CD74) ligand-receptor axis in necrotic myofibers from IMNM patients, suggesting the involvement of this signaling axis in immune-microenvironment crosstalk within IMNM-affected muscle. These observations stem from muscle-tissue transcriptomic and in situ staining analyses; no serum measurements or clinical correlation coefficients were available. Further functional experiments and external-cohort validations are warranted [31].

The Toll-like receptor (TLR) family serves as key sensors of danger signals and shows subtype-specific expression of TLR3/4/7/9 in IMNM. Data from an Italian single-center cohort including muscle-tissue specimens of 22 IMNM patients reveal increased TLR4 protein and transcript levels in IMNM muscle, with higher levels in anti-SRP than anti-HMGCR patients. Enhanced TLR4 signaling elicits robust innate immune responses to DAMPs, consistent with the phenotype of severe disease and prominent myonecrosis in anti-SRP patients. IMNM is characterized by elevated TLR7 transcription and reduced TLR9 transcription. TLR4 signaling drives pro-inflammatory M1 macrophage polarization—more striking in the anti-SRP subgroup—and M1 macrophages secrete pro-inflammatory mediators to aggravate muscle injury [33]. With respect to methodology, TLR4 protein was quantified by immunofluorescence using corrected total cell fluorescence (CTCF) measurement in ImageJ (version 1.54f, National Institutes of Health, Bethesda, MD, USA), and transcript (mRNA) levels were assessed by real-time quantitative PCR, normalized to the GAPDH reference gene and calculated by the 2^−ΔΔCt^ method. Between-subgroup differences were evaluated using the non-parametric Mann–Whitney U test; both protein and mRNA levels were significantly higher in anti-SRP than in anti-HMGCR samples (protein *p* = 0.0072; mRNA *p* = 0.0116). Of note, this cohort did not include seronegative IMNM subjects. The “high” versus “low” designations therefore reflect statistically significant between-subgroup differences rather than predefined clinical cut-off values.

Notably, multiple studies have validated the central role of necroptosis—a form of programmed necrosis mediated by the receptor-interacting protein kinase 1 (RIPK1)-receptor-interacting protein kinase 3 (RIPK3)-mixed lineage kinase domain-like protein (MLKL) necrosome—in myofiber injury in inflammatory myopathies. Data from a Chinese single-center retrospective cohort including 138 muscle biopsy specimens revealed a high p-MLKL-positive rate of 93.48% in IMNM muscle, with p-MLKL radiating from necrotic foci toward adjacent non-necrotic myofibers. However, p-MLKL can also be detected in other myopathies featuring myonecrosis with limited specificity; external cohorts and functional experiments are required for further validation. Another Chinese single-center study combining muscle biopsies (including 17 IMNM patients) and in vitro functional experiments in C2C12 myoblasts demonstrated overactivated necroptosis in muscle tissues from DM and IMNM patients. The expression of RIPK3, MLKL, and their phosphorylated effector molecules was elevated; protein levels were positively correlated with the degree of myonecrosis and serum muscle enzymes, and negatively correlated with MMT-8 muscle strength scores. MLKL colocalized with the DAMP molecule HMGB1 within necrotic myofibers, suggesting that necroptotic myofibers represent an important source of HMGB1. In vitro experiments confirmed that TNF-α could trigger MLKL-dependent necroptosis in myoblasts, and the inhibition of this pathway could attenuate cell death. This study is limited by its small sample size, lack of disease controls, and absence of external-cohort validation [34,35].

Based on this evidence, we propose a self-amplifying DAMP-necroptosis loop operating within IMNM-affected muscle: antibody-triggered myofiber necrosis releases DAMPs, which activate adjacent myofibers and infiltrating macrophages via pattern-recognition receptors such as TLR4. This in turn initiates RIPK3-MLKL-dependent necroptosis and aggravates tissue destruction. This vicious loop provides a mechanistic explanation for the extensive myonecrosis despite sparse lymphocytic infiltration.

### 4.3. Cytokine Axes: TWEAK/Fn14 and IL-6/STAT3 Trans-Signaling Axes

In 2023, Yang et al. reported that tumor necrosis factor-like weak inducer of apoptosis (TWEAK) and its receptor fibroblast growth factor-inducible 14 (Fn14) are markedly overexpressed in damaged muscle tissues from 37 IMNM patients. As the predominant innate immune cells within lesions, CD68^+^ macrophages secrete TWEAK, which binds to Fn14 on myofibers and activates downstream pro-inflammatory signaling cascades including nuclear factor kappa B (NF-κB). This further stimulates myofibers to produce multiple chemokines, such as monocyte chemoattractant protein-1 (MCP-1), interferon-inducible protein 10 (IP-10), and regulated upon activation normal T-cell expressed and secreted (RANTES), as well as pro-inflammatory cytokines including interleukin-1β (IL-1β) and IL-6. Fn14 expression levels are significantly correlated with the extent of myofiber necrosis and clinical muscle weakness. Its expression on regenerating myofibers also indicates that this pathway exerts complex regulatory effects in innate immunity-mediated tissue repair [36].

Studies have confirmed that the IL-6/signal transducer and activator of transcription 3 (STAT3) signaling axis represents another critical myogenic inflammatory amplification pathway in IMNM. Based on a single-center cohort consisting of 30 IMNM patients and 12 controls, integrating muscle immunohistochemistry, quantitative polymerase chain reaction (qPCR), serum cytokine profiling, and multiple in vitro experiments using human myoblasts, Ma et al. demonstrated prominent sarcoplasmic MCP-1 (CCL2) accumulation in IMNM muscle, which correlated positively with myofiber necrosis, regeneration, and the magnitude of inflammatory cell infiltration. IMNM patients exhibited elevated serum levels of IL-6, soluble interleukin-6 receptor (sIL-6R), and MCP-1; serum MCP-1 was higher during disease flares than in remission, positively associated with serum creatine kinase and lactate dehydrogenase, and inversely associated with MMT-8 muscle strength scores [37]. Human myoblasts predominantly express the co-receptor gp130 (IL-6ST) but barely express membrane-bound IL-6R, rendering them responsive primarily to IL-6 trans-signaling rather than classical IL-6 signaling. High serum levels of IL-6 form complexes with sIL-6R in IMNM patients, initiating IL-6 trans-signaling and subsequently inducing specific phosphorylation of the intracellular transcription factor STAT3. Mechanistically, phosphorylated STAT3 directly binds to the promoter region of the MCP-1 gene and drives its transcription in myoblasts; the knockdown of STAT3 largely abolishes MCP-1 induction triggered by the IL-6/sIL-6R complex. Secreted MCP-1 further recruits monocytes and macrophages into muscle lesions, amplifying local inflammatory responses and thereby participating in the immunopathogenesis of IMNM [37]. Nevertheless, both investigations are single-center studies with limited sample sizes; relevant findings await further validation in larger, multicenter cohorts and appropriate preclinical animal models.

### 4.4. Remodeling of Innate Immune Cell Subsets

Integrating single-cell RNA-seq reference datasets (6 IIM cases: 3 anti-MDA5-positive DM and 3 ASS; 3 normal controls) and deconvolution (CIBERSORTx) of bulk transcriptomic data from a large Chinese Han cohort (203 IIM including 37 IMNM and 19 normal controls), researchers in one cutting-edge study constructed muscle cellular atlases to dissect the immunopathogenesis of IMNM.

Dysregulated natural killer (NK) cells: Anti-SRP-positive IMNM patients exhibit characteristic functional skewing of NK cell subsets. The proportion of CD56dimCD16dim NK cells, which exert potent cytotoxicity as their primary function, is markedly elevated, whereas the percentage of CD56brightCD16bright NK cells responsible for immune regulation and maintenance of local immune homeostasis is significantly reduced (single-cell exploratory study) [31].

Enhanced dendritic cell activation: Conventional type 2 dendritic cells (cDC2) are enriched in IMNM muscle lesions (single-cell exploratory study). As pivotal myeloid antigen-presenting cells, cDC2 initiate downstream immune responses via chemokine signaling pathways and serve as a critical bridge linking innate immune priming and adaptive immunity [31].

Functional remodeling of macrophage subsets: Macrophages with high C1QC expression are highly enriched in IMNM muscle tissue, showing the highest abundance among all IIM subtypes. This subset specializes in antigen processing and presentation with robust phagocytic capacity; upon recognizing and engulfing necrotic myofibers, it further triggers innate immune responses and fuels the amplification of inflammatory cascades [31]. The frequency of LYVE1-positive macrophages declines in IMNM. Since this subset predominantly mediates tissue repair, neural development, and myelin sheath preservation, its depletion indicates impaired regenerative capacity and drives inflammation toward persistent tissue damage. Decision-tree models built upon this histologic marker and symptom duration achieved acceptable predictive accuracy for risk stratification, though these findings require prospective multicenter validation. Reduction in angiogenesis-related monocytes: serpin family B member 2 (SERPINB2)-positive monocytes responsible for angiogenesis are markedly decreased in the anti-SRP subgroup. The loss of this pro-angiogenic subset indicates severe innate immunity-mediated defects in tissue repair in IMNM, which may underlie the severe clinical manifestations, poor prognosis, and high relapse rate of this subtype [31]. In a single-center retrospective histopathological study of 62 IMNM patients, Sun et al. demonstrated that a high density of CD163^+^ macrophages localized within perimysial connective tissue represents an independent poor prognostic factor. Patients with abundant perimysial CD163^+^ macrophages were characterized by more severe clinical manifestations, higher inflammatory markers, and worse immunotherapy response. Unsupervised clustering revealed that cases with disease duration over 5 months tended to exhibit predominant CD163^+^ macrophage accumulation, implying a phenotypic shift in tissue-resident innate immune cells from compensatory repair toward pathological tissue remodeling [38].

### 4.5. Therapeutic Implications of Innate Immunity

**Figure 2:**
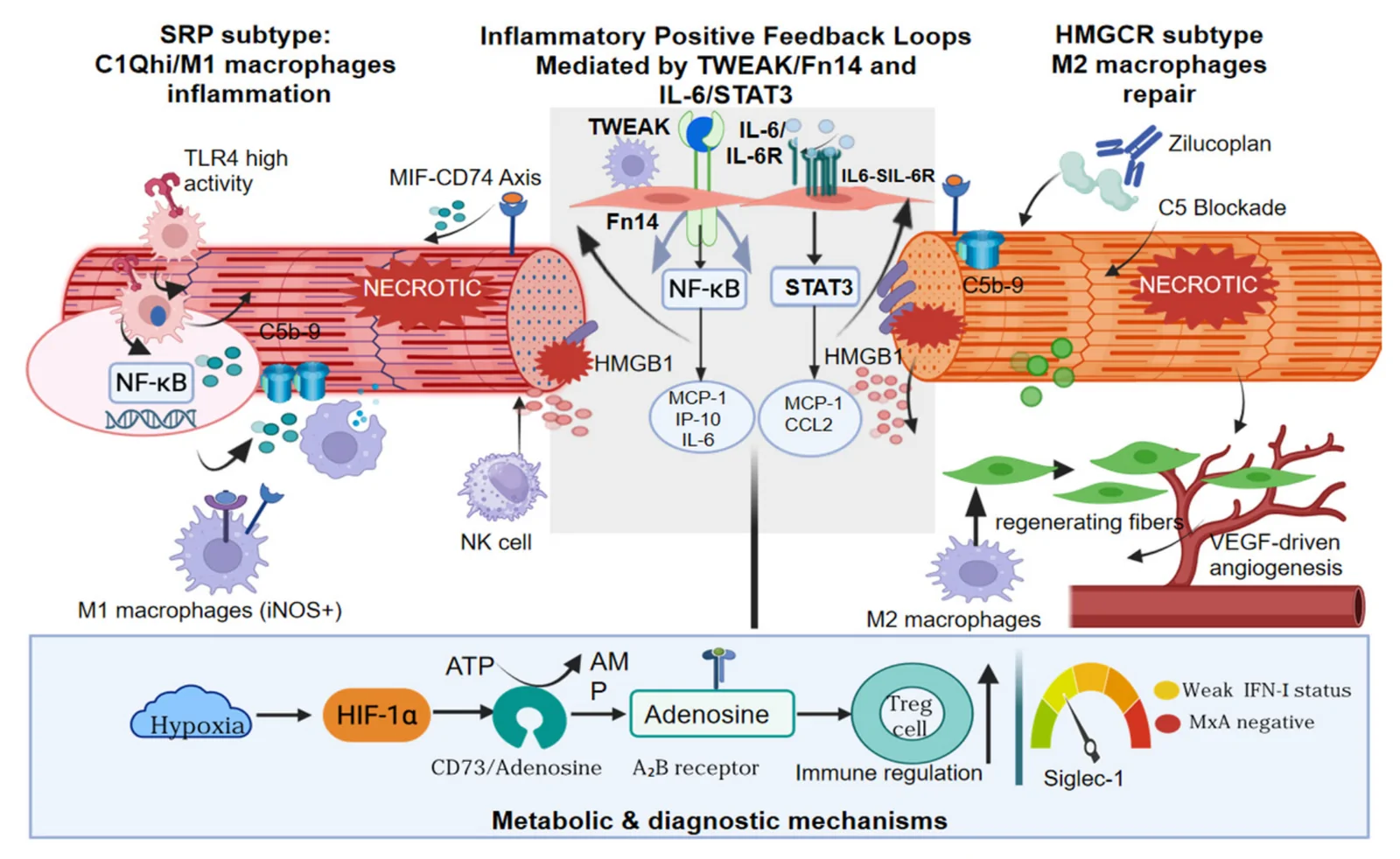
Distinct innate-immune signatures between anti-SRP and anti-HMGCR IMNM. Anti-SRP IMNM exhibits M1-macrophage-dominant pro-inflammatory responses with TLR4-NF-κB and NK-cell activation. TWEAK/Fn14 and IL-6/STAT3 are shared inflammatory positive-feedback axes for both subtypes, triggered by HMGB1 released from necrotic myofibers. Anti-HMGCR IMNM is characterized by reparative M2 macrophages, VEGF-driven angiogenesis and muscle regeneration. The hypoxia-HIF-1α-adenosine immune-regulatory axis and Siglec-1-based type-I-interferon status are shown at the bottom. Zilucoplan-mediated C5 blockade mitigates complement-dependent myofiber injury across both subtypes.

Fn14-neutralizing antibodies block TWEAK-Fn14 binding and suppress NF-κB activation without impairing the reparative functions of Fn14 [36]. IL-6 receptor inhibitors such as tocilizumab may block the IL-6/sIL-6R/STAT3 cascade [37]. However, these agents have not been evaluated in IMNM-specific RCTs, and their clinical efficacy remains unconfirmed. This model predicts that TLR4 antagonists may exert synergistic effects with B-cell-targeted therapy by simultaneously blocking both the initiation and amplification phases of pathogenesis. (Innate immune mechanisms are shown in Figure 2).

## 5. Cellular Immunity: Modulatory T-Cell Responses Within a Sparse Infiltrative Milieu

### 5.1. Cellular Immunity: Expression Patterns of MHC-I and ICAM-1

In a single-center retrospective biopsy cohort consisting of 357 muscle specimens (including 43 IMNM cases), Milisenda et al. reported universal MHC-I expression in IMNM myofibers, with 79% showing a focal sarcoplasmic staining pattern; MHC-I immunohistochemistry yielded a sensitivity of 0.95 and specificity of 0.82 for the diagnosis of idiopathic inflammatory myopathies [13]. This cytoplasmic retention pattern indicates that MHC-I upregulation in IMNM may result not only from secondary cytokine-driven responses but also from endoplasmic reticulum stress triggered by myofiber necrosis and regeneration [13]. Using automated quantitative morphometric immunofluorescence on muscle biopsies (7 IMNM among a total of 33 IIM cases), Nishimura et al. demonstrated that sarcolemmal levels of MHC-I, major histocompatibility complex class II (MHC-II), and intercellular adhesion molecule 1 (ICAM-1) in IMNM were lower compared with IBM and ASS. Endomysial vascular ICAM-1 expression in IMNM was comparable to normal controls, arguing against vasculitis and endothelial activation as initiating pathogenic factors [12]. Nevertheless, prominent CD4^+^ and CD8^+^ T-cell infiltration could still be detected within endomysial and perimysial compartments of IMNM muscle tissue, implying that T-cell recruitment can occur independently of pronounced endothelial ICAM-1 upregulation. Bulk transcriptomic data corroborated these protein-level observations, and IMNM showed the enrichment of gene pathways governing cell death and developmental regulation [12]. Analysis derived from deconvolution of the same single-cell atlas (Section 4.4) further reveals dynamic shifts in the T-cell repertoire, offering an intrinsic mechanistic explanation for the hypoactivated phenotype of IMNM. Studies demonstrate compensatory expansion of CD4^+^ regulatory T cells within muscle tissue, representing a negative feedback loop that restrains inflammation, while CD4^+^ central memory T cells are markedly reduced, indicating T-cell differentiation from an immune memory phenotype toward an effector phenotype [31].

Of note, in anti-HMGCR-positive IMNM, antigen-specific HMGCR-reactive CD4^+^ T cells skewed toward a Th1-Th17 phenotype have been identified in peripheral blood and affected skeletal muscle, with HMGCR-reactive TCR clonotypes enriched in target tissue despite sparse histological inflammation [39]. Even with these observations, it remains unresolved whether these tissue-resident T-cell populations act as primary disease drivers, inflammatory amplifiers, or merely secondary epiphenomena.

### 5.2. The Th1-M1 Axis and CD155-CD226/TIGIT Checkpoint Axis

In a retrospective biopsy-based study of 16 IMNM patients, eight non-immune-mediated necrotizing myopathy (nIMNM) patients, and five healthy controls, a prominent Th1-M1-polarized immune response was identified in IMNM lesions. Th1-derived interferon gamma (IFN-γ) upregulates myofiber MHC-I (consistent with the cytokine-driven MHC-I upregulation noted in Section 5.1) and drives classical M1-polarization of CD68^+^ macrophages via signal transducer and activator of transcription 1 (STAT1) signaling. Activated M1 macrophages in turn secrete IL-12 and TNF-α, further reinforcing Th1-cell differentiation and creating a positive-feedback inflammatory loop. Accordingly, the transcript levels of IFN-γ, TNF-α, IL-12, and STAT1 are markedly elevated in IMNM muscle compared with nIMNM and healthy controls. CD68^+^ macrophages are diffusely distributed across IMNM lesions rather than being restricted exclusively to necrotic foci, a feature not observed in toxic nIMNM. Of note, M1 effector molecules inducible nitric oxide synthase (iNOS) and COX-2 showed no significant difference between IMNM and nIMNM, indicating that macrophage effector function per se cannot discriminate the two entities. Th2-related mediators are barely detectable in IMNM. This study provides correlative pathological and transcriptomic evidence; direct functional proof for the causal pathogenic role of the Th1-M1 axis remains absent [20]. In addition, CD26 (DPP-4), a T-cell co-stimulatory molecule, accumulates within necrotic-inflamed regions of IIM muscle. Although IMNM exhibits comparatively low overall CD26 expression, CD26-mediated co-stimulation is hypothesized to amplify T-cell-derived pro-inflammatory cytokine production and exacerbate muscle-tissue injury [40]. Collectively, the Th1-M1 immune axis underpins the pattern of local tissue-targeted immune responses in IMNM and sustains disease progression via the amplification of intramuscular inflammation [20].

Dysregulation of the CD155-CD226/TIGIT checkpoint axis: In a single-center cohort consisting of 20 IMNM patients, CD155 was diffusely overexpressed on myofibers in 90% of cases. Infiltrating T cells exhibit enhanced CD226-mediated co-stimulation and attenuated TIGIT co-inhibition. The activation of this axis correlates with serum CK levels and muscle weakness [41].

### 5.3. T Cell Exhaustion: PD-1, TIM-3 and LAG-3

In retrospective analyses on frozen muscle biopsies (12 IMNM, 7 sporadic IBM, and 9 ICI-induced myositis patients; 8 non-diseased controls), researchers employed immunohistochemistry, immunofluorescence, and quantitative PCR to characterize the programmed death 1 (PD-1) pathway and markers associated with T-cell exhaustion at both protein and transcript levels. No molecular differences were identified between anti-SRP-positive and anti-HMGCR-positive IMNM subgroups within this cohort. These are purely correlative tissue-based observations, without supporting functional in vitro or in vivo mechanistic assays [42].

### 5.4. The CD73/ADA/A2B Metabolic-Immune Axis

Integrating cross-sectional human clinical biospecimens (64 serum samples, 63 peripheral-blood samples, and 34 skeletal-muscle biopsies from IIM patients including IMNM cases), experimental autoimmune myositis (EAM) mouse-model experiments, and in vitro T-cell stimulation assays, one study illustrated that ecto-5′-nucleotidase (CD73)/ADA-driven dysregulation of adenosine metabolism contributes to immune dysfunction in IMNM. Infiltrating macrophages upregulate CD73 to generate anti-inflammatory adenosine, yet abnormally elevated ADA accelerates adenosine degradation and abrogates the protective effects of adenosine. Defective A2B receptor signaling leads to hypoxia-inducible factor 1 alpha (HIF-1α) accumulation and STAT3 activation, which facilitates Th17 differentiation while suppressing Treg development. The activation of the A2B receptor downregulates HIF-1α (*p* = 0.0019), reduces p-STAT3^+^ Th17 cells (16.32% to 6.06%; *p* = 0.0025), and restores functional Tregs (0.45% to 2.89%; *p* < 0.0001) (experimental autoimmune myositis model, not validated in IMNM patients). These findings identify the A2B receptor as a promising metabolic therapeutic target [43].

### 5.5. The Role of T Cells in IMNM

Taken together, cumulative evidence favors the view that T-cell-driven cytotoxicity is unlikely to serve as a universal primary pathogenic driver for IMNM, although this cannot be fully excluded in certain autoantibody-defined subgroups such as anti-HMGCR-positive IMNM. Three complementary lines of evidence support this interpretation. First, IMNM-infiltrating T cells frequently display an exhausted PD-1^+^T cell immunoglobulin and mucin domain 3 (TIM-3)^+^ lymphocyte activation gene 3 (LAG-3)^+^ phenotype associated with limited cytotoxic potential, arguing against a dominant active cytotoxic program; it should be noted, however, that exhausted T cells may still retain partial pro-inflammatory function [42]. Second, type-I interferon signatures, the canonical molecular bridge connecting innate immune sensing to adaptive effector T-cell differentiation, are often low or absent in IMNM muscle lesions, though this pattern is not universal across all patient subgroups [13]. Third, clinical observations show that B-cell-depleting therapy and FcRn antagonism can induce clinical improvement in subsets of IMNM patients without directly targeting T cells. Still, treatment response is heterogeneous, and this observation alone cannot definitively establish that T-cell activation lies strictly downstream of humoral immune responses.

**Figure 3:**
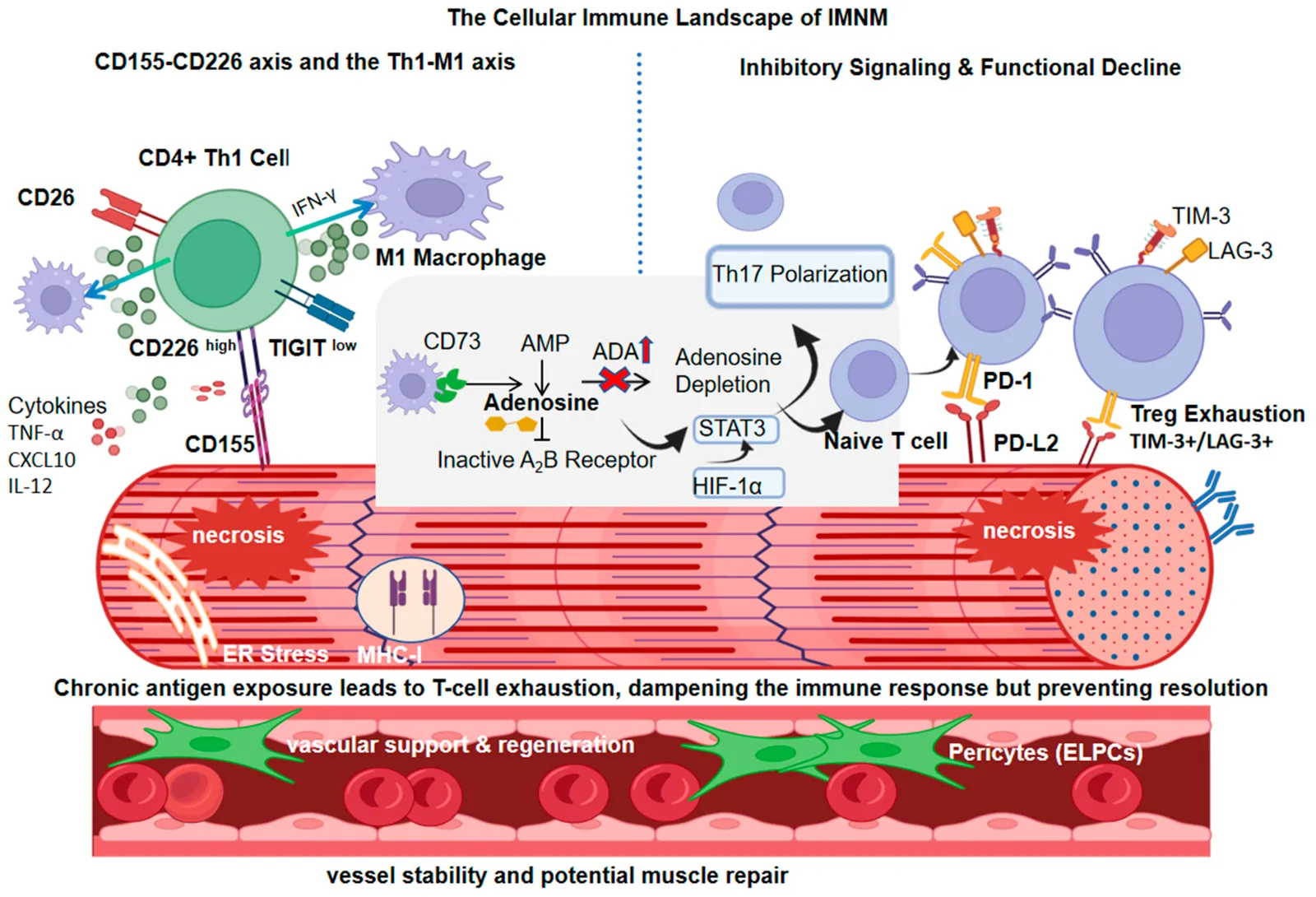
Cellular immune mechanisms in IMNM: the Th1-M1 axis, immune checkpoint disturbance, and adenosine metabolism, which collectively induce T cell exhaustion and secondary muscle damage under mild inflammation.

This reasoning does not render T cells functionally irrelevant: tissue-resident T cells likely act as local inflammatory amplifiers via Th1-M1 signaling, immune-checkpoint dysregulation, and metabolic-immune circuit disturbance, sustaining chronic intramuscular inflammation and potentially contributing to therapeutic resistance [20,43]. Collectively, T cells are best conceptualized as important pathological modulators rather than universal disease-initiating triggers in IMNM. (**Cellular immune mechanisms are shown in** Figure 3).

## 6. Failed Regeneration: From Necrosis to Disability

### 6.1. Vascular Barrier Disruption and Microcirculatory Failure

Single-cell analysis reveals a marked reduction in hypoxia-inducible factor 3 alpha (HIF3A^+^) endothelial cells (which maintain endothelial barriers) and platelet-derived growth factor receptor beta (PDGFRB^+^) pericytes in IMNM. The collapse of this physical barrier creates a permissive pathway for autoantibody extravasation and transendothelial migration of highly cytotoxic NK cells. Although compensatory proliferation of endothelial-like pericytes (ELPCs) emerges in response to myonecrosis, the absence of pro-angiogenic SERPINB2^+^ monocyte support (as noted in Section 4.4), coupled with pathological hyperplasia of vascular smooth muscle cells (VSMCs), especially prominent in patients with the anti-SRP subtype, results in failed reconstruction of functional microcirculation [31].

### 6.2. Impaired Myofiber Regeneration

Compared with ASS, IMNM exhibits milder loss of type I myofibers. Necrotic myofibers display robust activation of the MIF-CD74 axis, which recruits inflammatory cells and establishes a self-sustaining cycle of inflammatory infiltration, necrosis, and further immune recruitment. Myogenic progenitor cells (MPCs) are slightly expanded yet insufficient to offset tissue damage, indicating that immune-mediated injury overwhelms endogenous regenerative capacity. The anti-HMGCR subtype manifests active reparative responses; nevertheless, young patients develop refractory disease accompanied by sluggish regeneration, reflecting an imbalance between tissue injury and repair. Capillary density is elevated in anti-HMGCR lesions (303 ± 27/mm^2^ versus 204/mm^2^) (small-sample study), driven by HIF-1α^+^ pro-angiogenic macrophages that upregulate vascular endothelial growth factor A (VEGF-A) and C-X-C motif chemokine ligand 12 (CXCL12) (correlation between VEGF-A^+^M2 macrophages and capillaries: rs = 0.98; *p* = 0.0004). In contrast, anti-SRP lesions lack pro-angiogenic macrophages and feature suppressed angiogenesis [44].

### 6.3. Macrophage-Mediated Repair Processes

Macrophage-mediated repair processes exert dual effects. Spatial pathological studies demonstrate that M2-type macrophages participate in tissue repair, yet excessive perimysial CD163^+^ macrophage infiltration, which marks a shift from compensatory repair toward pathological remodeling and poor prognosis, is detailed in Section 4.4. [38]. The anti-SRP-positive subtype lacks accumulation of pro-angiogenic macrophages, leading to suppressed angiogenesis. This finding is consistent with the SERPINB2^+^ monocyte depletion noted in Section 4.4 and accounts for the disparities in pathological compensation and tissue repair capacity between the two IMNM subgroups [31]. As a compensatory response to necrosis, macrophages attempt to balance the local microenvironment via the CD73/adenosine deaminase (ADA) pathway; however, overexpressed ADA abrogates the protective effects of adenosine and further disrupts microenvironmental homeostasis [43].

### 6.4. Systemic Immune Profiles and Emerging Biomarkers

Machine learning classifies IMNM muscle biopsies with an accuracy exceeding 91% using pooled retrospective biopsy samples collected across multiple sites in the United States and Spain, without external independent validation, and apolipoprotein A4 (APOA4) is identified as a distinctive signature gene for anti-HMGCR IMNM. Muscle transcriptomics reveals no dominant Th1/Th2/Th17 signature; high SPP1 expression reflects tissue repair and debris clearance rather than immune-mediated cytotoxic attack [45]. Serum growth differentiation factor 15 (GDF15) is markedly upregulated and colocalizes with regenerating myofibers. CXCL10 is moderately elevated in IMNM (425 ± 324 pg/mL; cut-off value: 180 pg/mL; sensitivity = 0.80; specificity = 0.71; derived from a combined inflammatory myopathy cohort including IMNM and sporadic IBM; single-center retrospective cohort from Ghent University Hospital, Belgium, not independently validated), which contrasts with the drastically increased CXCL10 levels observed in inclusion body myositis (IBM) [46]^.^ Among the 27 cytokines measured, 20 were significantly elevated in the serum of IMNM patients. IP-10 (5709 pg/mL) and MCP-1 (80.1 pg/mL) distinguish IMNM from other neurological disorders within a Japanese single-center cohort, and external validation datasets are lacking. Interleukin-1 receptor antagonist (IL-1Ra) is the only factor positively correlated with the recovery of muscle function [45]. Despite these advances, no single biomarker possesses sufficient specificity for differential diagnosis and patient stratification. For potential clinical application, the best-supported findings from existing studies are circulating CXCL10 (IP-10) and GDF15 for diagnostic support (GDF15 threshold 300 pg/mL for combined interpretation without reported standalone sensitivity and specificity); IP-10 and MIP-1α for longitudinal disease-activity monitoring (both correlate with serum CK and decline after immunosuppressive therapy); MCP-1 to help distinguish IMNM from inclusion body myositis in research-based cohorts; and interleukin-7 (IL-7) to discriminate anti-SRP from anti-HMGCR disease based on a small-sized Italian single-center cohort; IL-1Ra has additionally been associated with functional recovery. These cytokines should be regarded as research-only adjuncts to, rather than replacements for, autoantibody testing and CK measurement. None of these biomarker findings have been replicated in large-scale, prospective, multicenter cohorts [33].

## 7. Anti-SRP Versus Anti-HMGCR: One Autoantibody Class, Two Distinct Disease Phenotypes

### 7.1. Shared Core Mechanisms and Subtype-Specific Pathological Heterogeneity in IMNM

Although all three subtypes of immune-mediated necrotizing myopathy (IMNM) share a core pathogenic cascade initiated by autoantibodies and amplified by damage-associated molecular patterns (DAMPs), they exhibit substantial divergence in downstream effector mechanisms. Pathological features common to all subtypes are as follows: autoantibodies trigger myofiber injury via complement-dependent and internalization pathways; released DAMPs engage pattern recognition receptors; the inflammatory infiltrate is predominantly composed of M1/M2 macrophages; and widespread T-cell exhaustion is observed across all groups.

Disparities between anti-SRP and anti-HMGCR subtypes cannot be fully explained by antibody properties alone, as both autoantibodies bind myofibers, activate complement, and undergo internalization. Comparative analyses uncover consistent differences in innate immune activation: TLR4 expression is higher in anti-SRP muscle tissues (*p* = 0.0116), which drives more robust M1 macrophage polarization [33]. The macrophage landscape differs fundamentally: anti-SRP lesions are dominated by iNOS^+^ M1 macrophages with suppressed angiogenesis, whereas anti-HMGCR lesions harbor VEGF-A^+^ M2 macrophages that boost capillary proliferation (303 ± 27 versus 204/mm^2^; correlation between VEGF-A^+^ M2 macrophages and capillaries: rs = 0.98; *p* = 0.0004) [44]. Distinctive B-cell lymphoma 2 (Bcl-2^+^) C-C chemokine receptor type 4 (CCR4^+^) lymphoid aggregates emerge as a hallmark of anti-HMGCR IMNM (observed in 26.3% of cases) (single-center cohort), creating an anti-apoptotic microenvironment [47]. Pro-angiogenic SERPINB2^+^ monocytes are depleted in anti-SRP patients (36.6% versus 46.7%) (single-cell exploratory cohort, not independently replicated), consistent with impaired tissue repair.

Notably, the three IMNM subtypes exhibit unique phenotypic discrepancies: anti-SRP IMNM is characterized by increased TLR4 expression, prominent M1 macrophage polarization, inhibited angiogenesis, and reduced pro-angiogenic SERPINB2^+^ monocytes; anti-HMGCR IMNM presents abundant VEGF-A^+^ M2 macrophages that facilitate capillary proliferation and specific Bcl-2^+^CCR4^+^ lymphoid aggregates; while seronegative IMNM features decreased sarcolemmal MAC deposition and tended to show better treatment responses (87.5% vs. 61%, *p* = 0.0641, in a single Chinese cohort). Accordingly, the hierarchical pathogenic cascade summarized in this review serves as a universal foundational framework overlying diverse subtype-specific pathological alterations, rather than a uniform mechanism identically applicable to all IMNM subgroups (Table 1).

### 7.2. Genetic Susceptibility

Genetic susceptibility plays a critical role in the pathogenesis of IMNM. The strongest genetic association in anti-HMGCR IMNM is with human leukocyte antigen (HLA-DRB1*11:01), carried by approximately 78% of patients (versus 17% of healthy controls), implying a role for shared antigen presentation in the breakdown of B-cell tolerance [48]. By contrast, anti-SRP IMNM is associated with distinct HLA alleles—DRB1*08:03 (in Japanese populations), B*50:01, and DQA1*01:04. These HLA associations have two mechanistic implications. First, specific HLA class II molecules may preferentially present SRP54 or HMGCR peptides to CD4^+^ T cells, providing the T-cell help required for antibody class switching. Second, the remarkably high odds ratio (OR > 30) for DRB1*11:01 in anti-HMGCR IMNM represents one of the strongest HLA-disease associations among autoimmune disorders. Despite this robust genetic linkage, environmental triggers remain unidentified, and the clinical utility of HLA genotyping in IMNM has not yet been investigated [49].

### 7.3. Malignancy Risk Across IMNM Subtypes

Malignancy risk differs markedly across IMNM subtypes. Although classically emphasized for anti-HMGCR disease, the strongest cancer association is actually observed in seronegative IMNM: in a 115-patient cohort, the standardized incidence ratio (SIR) was highest in seronegative disease (8.35), exceeding that of anti-HMGCR IMNM (2.79), whereas anti-SRP IMNM showed no significant increase (1.65) [50]. However, this gradient derives from a single French cohort with a limited seronegative subgroup, and subsequent studies reached discordant conclusions: a Mayo Clinic matched case–control study found no significant difference in malignancy prevalence among the three subtypes (*p* = 0.34), and a large Johns Hopkins cohort referenced to the US SEER population found no significant increase in cancer risk for either anti-HMGCR (SPR 1.35) or anti-SRP (SPR 0.71) IMNM [51,52]. The 2023 IMACS international guideline classifies anti-HMGCR as a moderate-risk and anti-SRP as a low-risk factor but, owing to heterogeneous antibody testing, does not stratify seronegative IMNM and acknowledges that its recommendations lack level-A evidence and rely substantially on expert consensus. In anti-HMGCR IMNM, the overall prevalence of concomitant cancer ranges from 20% to 25%, with a 2–3-fold elevated cancer risk. Risk stratification by statin exposure reveals that statin-naive patients have a malignancy rate of 25–35%, whereas only 5–10% of statin-exposed patients develop cancer. Independent risk factors include age over 50 years, male sex, and no prior statin exposure. Malignancies are usually detected within 1 to 3 years after IMNM onset, and autoantibody titers decline following successful anti-tumor therapy, supporting a paraneoplastic mechanism [53]. Cancer screening recommendations vary across guidelines and are not fully standardized; current consensus generally recommends comprehensive screening for anti-HMGCR-positive patients, with intensified surveillance for those aged over 50 years and statin-naive individuals. The most frequently associated malignancies consist of lung cancer, breast cancer, gastrointestinal cancers, and hematological malignancies [50,54]. It should be emphasized that this malignancy association is established for clinically definite IMNM (whether anti-HMGCR or seronegative)—that is, patients presenting with muscle weakness—and has not been demonstrated for incidental seropositivity in asymptomatic individuals or in patients without myopathy. Because autoantibody testing is clinically indicated in the setting of suspected myopathy, isolated antibody detection in a truly asymptomatic person is uncommon, and its prognostic significance remains unstudied; current screening recommendations therefore apply to patients with anti-HMGCR IMNM rather than to incidental seropositivity alone, and no consistent cancer association has been reported for anti-SRP antibodies.

### 7.4. Subtype-Specific Therapeutic Strategies

Anti-SRP and anti-HMGCR IMNM exhibit distinct innate immune profiles, and individualized therapeutic strategies may be inferred from current mechanistic evidence. Theoretically, anti-SRP patients, characterized by TLR4-driven M1 macrophage polarization and suppressed angiogenesis, may benefit from a combination of TLR4 antagonism and VEGF-pathway augmentation. Anti-HMGCR patients, featuring M2-mediated angiogenesis and Bcl-2-positive lymphoid aggregates, may respond better to B-cell depletion, whereas complement inhibition has not proven effective. Seronegative IMNM patients show less membrane attack complex deposition and tend to show better treatment responses (87.5% vs. 61%, *p* = 0.0641, in a single Chinese cohort), and generally require only mild immunosuppression [9]. Statin-naive anti-HMGCR patients carry a 25–35% malignancy risk and warrant intensified cancer surveillance.

Based on the above subtype-specific pathogenic mechanisms, early therapeutic intervention is theoretically warranted. Immunosuppressive therapy should be initiated before extensive, irreversible myofiber necrosis and muscle atrophy emerge to block the amplification of muscle injury and avoid permanent muscle weakness. To date, no evidence-based guidelines for IMNM have established standardized protocols for “early intervention” or “combination therapy targeting amplification pathways”. Current clinical regimens are predominantly derived from retrospective cohorts and case series [55]. In routine clinical practice, first-line treatment consists of high-dose glucocorticoids, which may be combined with intravenous immunoglobulin (IVIg) as needed. Rituximab is often administered early to patients with refractory disease or anti-SRP-positive IMNM. Conventional combination regimens combine glucocorticoids with non-biologic immunosuppressants such as tacrolimus, methotrexate, or mycophenolate mofetil, and IVIg can be supplemented for severe cases. The “combination therapy targeting amplification pathways” proposed in this review is derived from the aforementioned pathogenic cascade and adopts a dual therapeutic strategy: eliminating pathogenic autoantibodies to halt disease initiation, while suppressing immune amplification loops to abrogate sustained myocyte damage. Notably, this novel combinatorial approach is merely a mechanistic hypothesis lacking validation from prospective clinical trials; it is not an established routine therapeutic regimen in clinical practice.

Several of the pathways discussed above already have candidate agents under active investigation. The humoral axis is the most clinically advanced: the FcRn antagonist efgartigimod restores muscle function in humanized IMNM models, CD19-targeted CAR-T is being evaluated in the RESET-Myositis phase I/II trial, and anti-BCMA CAR-T achieved sustained serological remission in a refractory anti-SRP case [27,29,30].

**Figure 4:**
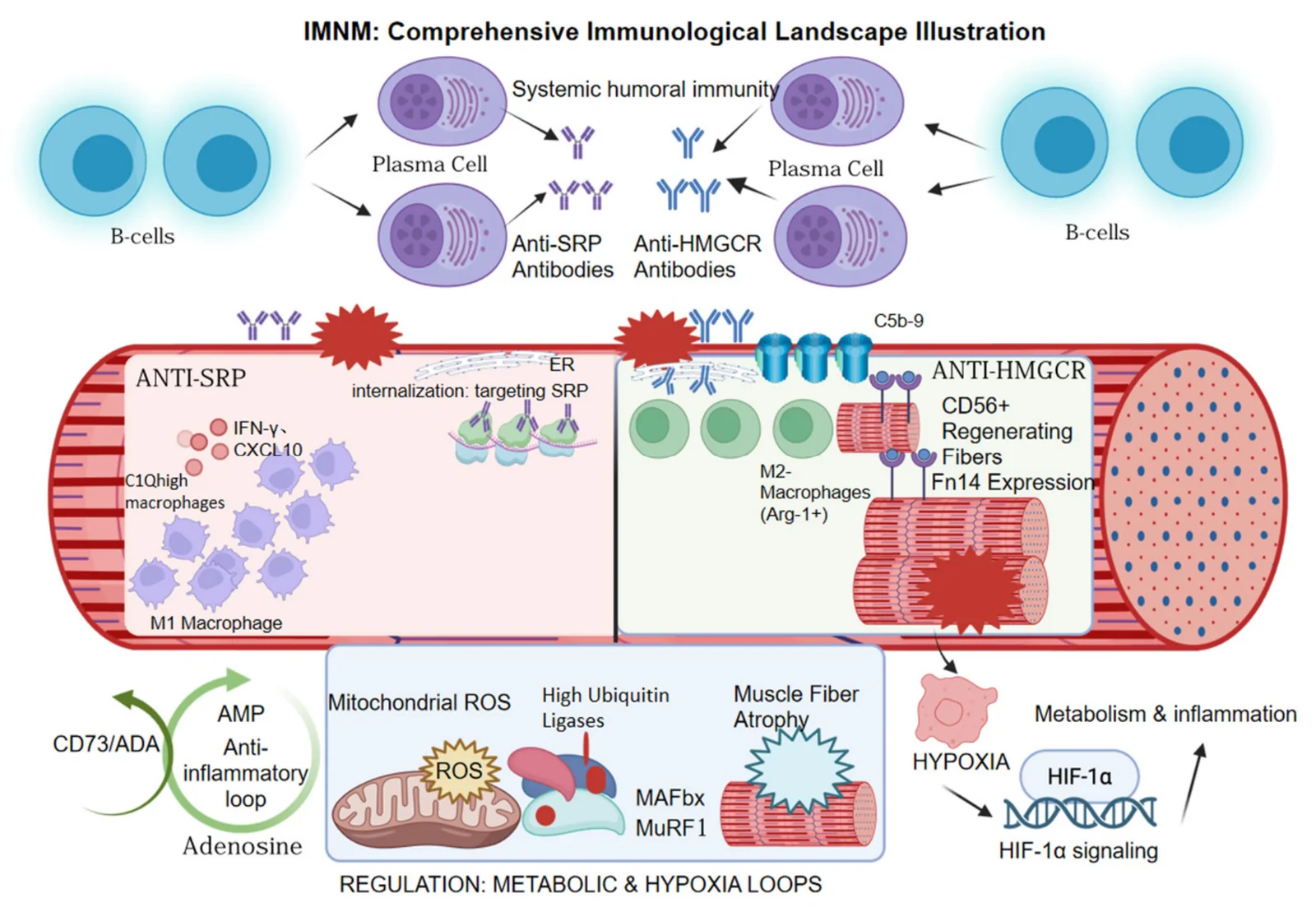
Comprehensive immunological landscape of IMNM. B-cell-derived plasma cells produce anti-SRP and anti-HMGCR autoantibodies to initiate complement-related myofiber damage. Anti-SRP IMNM exhibits C1q-high, M1-macrophage-predominant inflammation with increased IFN-γ and CXCL10, whereas anti-HMGCR IMNM shows reparative Arg-1^+^ M2 macrophages and CD56^+^ regenerating myofibers. Shared metabolic-hypoxia circuits in both subtypes comprise the CD73/ADA-adenosine axis, HIF-1α signaling, mitochondrial ROS, and ubiquitin-ligase-driven muscle atrophy.

Complement blockade with the C5 inhibitor zilucoplan failed its primary endpoint (change in serum creatine kinase at week 8) in a phase II randomized controlled trial of 27 patients, reinforcing the need for combined rather than isolated targeting. Interpreting this null result is complicated by several factors: (i) the trial enrolled patients with established active disease, whereas complement inhibition may be primarily preventive; (ii) the mixed anti-HMGCR and anti-SRP cohort, largely refractory and previously treated, may differ in complement dependence; (iii) the 8-week creatine kinase endpoint may not capture structural or functional change; (iv) established fatty replacement and fibrosis are irreversible; and (v) a complement-independent DAMP loop may bypass C5 blockade [55]. Several innate-immunity-directed candidate agents, such as TWEAK/Fn14-neutralizing antibodies, TLR4 antagonists, and modulators of the A2B adenosine receptor, have been supported by preclinical and mechanistic evidence from related disease models. IL-6 receptor inhibitors such as tocilizumab have shown preliminary efficacy in open-label pilot studies for refractory IMNM, yet large-scale randomized controlled trials remain absent [56]. However, there are no IMNM-specific randomized trials to confirm the clinical benefits of other innate-immunity-targeted therapeutics. Inhibitors targeting necroptosis (RIPK1/RIPK3/MLKL) and interventions aiming to boost VEGF-pathway activity are still at preclinical or conceptual stages. Accordingly, therapeutic research focusing on the humoral immune axis is proceeding vigorously, whereas innate immune amplification loops represent the most promising therapeutic frontier with the lowest level of clinical validation [34]. (The immune landscape is illustrated in Figure 4).

## 8. Conclusions

In this review, we comprehensively delineate the multi-layered immune mechanisms underlying IMNM and establish a hierarchical pathogenic framework. Anti-SRP and anti-HMGCR autoantibodies act as the primary initiators of disease, as supported by passive transfer experiments, clinical correlations between antibody levels and disease activity, and therapeutic responses to B-cell-targeted therapies. Innate immune cascades, including complement activation, DAMP release, TLR4 signaling, the TWEAK/Fn14 axis, and the IL-6/STAT3 pathway, sustain inflammatory amplification, and differential activation of these pathways directly accounts for subtype-specific pathological phenotypes. Cellular immunity plays a supporting role, while aberrant vascular remodeling and impaired muscle regeneration ultimately determine patients’ long-term functional outcomes.

Synthesizing available evidence, in this paper, we put forward five core hypotheses. First, IMNM represents an autoantibody-initiated and DAMP-amplified myofiber necrotizing disorder. Autoantibodies directly damage muscle fibers, and DAMPs released from necrotic cells activate innate immune receptors to form a complement-independent, self-perpetuating inflammatory loop, which also explains the sparse lymphocytic infiltration observed pathologically. Second, the disappointing clinical trial outcomes of complement inhibitors may partly reflect the possibility that complement primarily triggers the initial phase of necrosis. Disease persistence and progression rely on the subsequent DAMP cascade, so isolated complement blockade may yield limited efficacy. This necessitates early intervention or combination therapy targeting amplification pathways. Third, the clinical disparities between anti-SRP and anti-HMGCR IMNM do not arise from the antibodies themselves but from the divergent innate immune responses they elicit. Anti-SRP disease favors pro-inflammatory M1 polarization and suppressed angiogenesis, whereas anti-HMGCR disease is characterized by M2-mediated tissue repair and enhanced capillary proliferation. Such divergent phenotypes may stem from distinct cell death modalities and damage-associated signaling signatures induced by the two autoantibodies. Fourth, anti-HMGCR IMNM exhibits substantial heterogeneity. Both statin-exposure-induced conformational protein changes and tumor-derived cross-reactive antigen presentation can trigger autoantibody production against a shared HLA-DRB1*11:01 susceptibility background, leading to variable prognoses and treatment responses within this subtype. Fifth, IMNM lacks sustained type I IFN elevation, features predominantly exhausted T cells, and is dominated by macrophage-mediated clearance of necrotic tissue, demonstrating that it is neither an IFN-driven disorder nor a T-cell-mediated autoimmune injury. Accordingly, JAK inhibitors used for dermatomyositis and T-cell modulating therapies designed for inclusion body myositis lack mechanistic rationale for application in IMNM. Specifically, “early intervention” denotes initiating immunotherapy promptly after diagnosis, before irreversible fatty replacement and fibrosis develop, and “combination therapy” pairs an autoantibody-suppressing agent with complement blockade and blockade of the amplification loop, as detailed in Section 7.4.

All these hypotheses remain to be experimentally validated. Future research priorities can be structured into four translational directions. First, regarding clinically actionable pathways, combinatorial blockade experiments are required to dissect the respective pathogenic contributions of the DAMP-mediated necroptosis loop and complement activation during the early phase of IMNM. Second, optimized animal models should be established, including humanized and passive transfer models for anti-SRP and anti-HMGCR IMNM, while seronegative IMNM can be investigated using autoimmune myositis mouse models; [22] sequential sampling at early and late disease stages is essential to capture dynamic pathological changes. Third, biomarker validation based on independent multicenter longitudinal cohorts is warranted to confirm the diagnostic and disease-activity values of CXCL10/GDF15, as well as the subtype-specific signatures including TLR4, VEGF-A, SERPINB2, and Bcl-2^+^CCR4^+^. Fourth, prospective clinical studies with subtype-stratified randomized controlled trials are needed to compare the therapeutic efficacy of early intervention, late intervention, and multi-pathway combination regimens, with particular inclusion of seronegative IMNM patients.

The progressive adoption of multi-omics sequencing, preclinical animal models, and biomarker-stratified clinical trials will further elucidate the full pathogenesis of IMNM and lay a foundation for mechanism-guided individualized immunotherapy. The hierarchical model proposed herein encompasses autoantibody initiation, innate immune amplification, auxiliary cellular immunity, and impaired muscle regeneration. It serves as a theoretical roadmap for mechanistic investigations and a guiding framework for novel drug development, highlighting that durable disease control requires synergistic targeting of multiple critical nodes along the pathogenic cascade.

## Figures and Tables

**Table 1:** Comparison of clinical, serological, and histopathological features among anti-SRP-positive, anti-HMGCR-positive, and seronegative immune-mediated necrotizing myopathy (IMNM).

Feature	Anti-SRP-Positive IMNM	Anti-HMGCR-Positive IMNM	Seronegative IMNM
Age of onset and sex	Adults aged 40–50 years; female predominance (female:male ≈ 1.6–3.6:1)	Bimodal distribution; older-onset disease is often statin-associated, whereas adolescent/young-adult onset is usually statin-naive	No uniform characteristic established
Environmental triggers	Unclear	Marked geographic disparity: statin exposure predominates in European/American older patients, whereas a substantial proportion of Asian patients have no statin history	Definitive trigger unclear; malignancy is an important comorbidity
Clinical phenotype (muscle)	Mostly acute/subacute (a minority chronic and insidious); symmetric proximal weakness, lower-limb predominant; prominent cervical, bulbar and respiratory involvement; muscle atrophy in later stages; markedly elevated CK	Acute to subacute onset with prominent proximal weakness; dysphagia is common; markedly elevated CK	Limited descriptions; phenotype resembles antibody-positive IMNM; myalgia is the predominant symptom in Chinese patients
Clinical phenotype (extramuscular)	Interstitial lung disease in ~20%; subclinical myocardial injury may occur	Interstitial lung disease in <5%; cardiac involvement rare	Usually no obvious extramuscular manifestations
Core pathological changes	More severe necrosis and regeneration; C5b-9 positivity markedly lower than anti-HMGCR IMNM; scattered MHC-I-positive fibers	More frequent inflammatory infiltration, often perivascular; clustered MHC-I expression; MHC-II expression on fibers	Extensive myonecrosis and regeneration; sarcolemmal MAC may be present
Humoral immunity—Pathogenic antibodies	Anti-SRP autoantibodies	Anti-HMGCR autoantibodies	No IMNM-specific serum autoantibody
Humoral immunity—Complement-dependent injury	Complement can be activated, but sarcolemmal MAC (C5b-9) deposition is sparse	Sarcolemmal MAC deposition is more common; complement pathway prominently involved	Origin of humoral initiation unclear
Humoral immunity—Antibody internalization injury	Antibodies enter myocytes, inhibit protein transport; upregulate MAFbx/MuRF1 and induce atrophy; generate ROS and inhibit myoblast fusion/regeneration	Antibodies enter myocytes and disrupt cholesterol metabolism; similarly induce atrophy, elevate ROS and impair muscle repair	Origin of humoral initiation unclear
Humoral immunity—Local B cells/plasma cells	Intramuscular B-cell/plasma-cell infiltration is sparse; antibodies mainly derive from peripheral secondary lymphoid organs	Bcl-2^+^CCR4^+^ lymphoid aggregates in 26.3% of cases; more prominent local B-cell response	–
Humoral immunity—Therapeutic implications	B-cell depletion and FcRn antagonists are relatively effective; complement monotherapy has limited benefit	B-cell depletion is relatively effective; complement inhibition unproven (zilucoplan phase 2 negative)	–
Innate immunity—TLR expression	TLR4 expression markedly elevated, strongly driving inflammation	TLR4 expression relatively lower	–
Innate immunity—Macrophage polarization	Dominated by iNOS^+^ M1 pro-inflammatory macrophages; C1QC-high macrophages phagocytose necrotic fibers	Dominated by VEGF-A^+^ M2 reparative macrophages promoting angiogenesis	–
Innate immunity—NK and dendritic cells	Cytotoxic CD56dimCD16dim NK cells increased; cDC2 dendritic cells activated	No prominent NK-cell abnormality; cDC2 activation present	–
Cellular immunity	Th1-M1 axis; T-cell exhaustion; CD155-CD226	Th1-M1 axis; T-cell exhaustion; CD155-CD226	–
Regeneration	Pro-angiogenic SERPINB2^+^ monocytes markedly depleted; defective vascular repair	HIF-1α^+^ M2 macrophages secrete VEGF-A; increased capillary density (303 ± 27/mm^2^) with active compensatory repair; young patients may still show delayed regeneration	–
Cancer risk	Low-risk factor	Moderate-risk factor (IMACS); ~20–25% concomitant cancer with 2–3-fold elevated risk; cancer screening recommended	Highest SIR (8.35) in a single French cohort; not confirmed in subsequent cohorts
Genetic susceptibility loci	HLA-DRB1*08:03, B*50:01, DQA1*01:04	HLA-DRB1*11:01	No susceptibility gene established
Current treatment	Glucocorticoids combined with second-line immunosuppressants; rituximab	Discontinue and avoid the offending statin; steroids, methotrexate, azathioprine, mycophenolate or rituximab; IVIg is the most effective add-on for refractory disease	Glucocorticoids combined with second-line immunosuppressants
Prognosis	Only ~half achieve near-complete/complete strength recovery at 4 years; younger onset has more severe weakness; MRI shows more atrophy/fatty replacement than anti-HMGCR	Worse prognosis in patients under 50 years	Cancer risk elevated (SIR 8.35, one cohort); patients with synchronous cancer have shortened survival

Abbreviations: CK, creatine kinase; HMGCR, 3-hydroxy-3-methylglutaryl-coenzyme A reductase; MAC, membrane attack complex; MHC, major histocompatibility complex; MRI, magnetic resonance imaging; ROS, reactive oxygen species; SRP, signal recognition particle.

## Data Availability

No new data were created or analyzed in this study. Data sharing is not applicable to this article.

## Abbreviations

**Abbreviations**	**Full English Name**
IIM	Idiopathic Inflammatory Myopathy
IMNM	Immune-Mediated Necrotizing Myopathy
NAM	Necrotizing Autoimmune Myopathy
DM	Dermatomyositis
PM	Polymyositis
IBM	Inclusion Body Myositis
ASS	Antisynthetase Syndrome
ENMC	European Neuromuscular Centre
EULAR	European Alliance of Associations for Rheumatology
ACR	American College of Rheumatology
SRP	Signal Recognition Particle
HMGCR	3-Hydroxy-3-Methylglutaryl-CoA Reductase
MHC-I	Major Histocompatibility Complex Class I
MHC-II	Major Histocompatibility Complex Class II
ICAM-1	Intercellular Adhesion Molecule 1
MAC	Membrane Attack Complex (C5b-9)
RCD	Regulated Cell Death
DAMP	Damage-Associated Molecular Pattern
RIPK1	Receptor-Interacting Protein Kinase 1
RIPK3	Receptor-Interacting Protein Kinase 3
MLKL	Mixed Lineage Kinase Domain-Like Protein
p-MLKL	Phosphorylated MLKL
HMGB1	High Mobility Group Box 1
MIF	Macrophage Migration Inhibitory Factor
TWEAK	TNF-Like Weak Inducer of Apoptosis
Fn14	Fibroblast Growth Factor-Inducible 14
NF-κB	Nuclear Factor Kappa B
STAT3	Signal Transducer and Activator of Transcription 3
MCP-1	Monocyte Chemoattractant Protein 1
RANTES	Regulated upon Activation, Normal T cell Expressed and Secreted
IL-1β	Interleukin 1 Beta
IL-6	Interleukin 6
IL-7	Interleukin 7
sIL-6R	Soluble IL-6 Receptor
TNF-α	Tumor Necrosis Factor Alpha
IFN-γ	Interferon Gamma
CXCL10	C-X-C Motif Chemokine Ligand 10
CXCL13	C-X-C Motif Chemokine Ligand 13
ISG15	Interferon-Stimulated Gene 15
SIGLEC1	Sialic Acid Binding Ig-Like Lectin 1
SPP1	Secreted Phosphoprotein 1 (Osteopontin)
GDF15	Growth Differentiation Factor 15
TLR	Toll-Like Receptor
FPR1	Formyl Peptide Receptor 1
FcRn	Neonatal Fc Receptor
BCMA	B Cell Maturation Antigen
Th1	T Helper Type 1
Th17	T Helper Type 17
Treg	Regulatory T Cell
NK	Natural Killer Cell
DC	Dendritic Cell
MPC	Muscle Progenitor Cell
ELPC	Endothelial-Like Pericyte
PD-1	Programmed Death 1
PD-L2	Programmed Death Ligand 2
TIM-3	T Cell Immunoglobulin and Mucin Domain 3
LAG-3	Lymphocyte Activation Gene 3
TIGIT	T Cell Immunoreceptor with Ig and ITIM Domains
ADA	Adenosine Deaminase
CD73	Ecto-5′-Nucleotidase
DPP-4	Dipeptidyl Peptidase 4 (CD26)
CK	Creatine Kinase
LDH	Lactate Dehydrogenase
MMT-8	Manual Muscle Testing 8
MYOACT	Myositis Disease Activity Assessment
MDI	Myositis Damage Index
HAQ	Health Assessment Questionnaire
RCT	Randomized Controlled Trial
ROC	Receiver Operating Characteristic
AUC	Area Under the Curve
OR	Odds Ratio
CI	Confidence Interval
IHC	Immunohistochemistry
ELISA	Enzyme-Linked Immunosorbent Assay
qPCR	Quantitative Polymerase Chain Reaction
ChIP	Chromatin Immunoprecipitation
scRNA-seq	Single-Cell RNA Sequencing
DEG	Differentially Expressed Gene
PPI	Protein–Protein Interaction
RWR	Random Walk with Restart
VEGF	Vascular Endothelial Growth Factor
HIF-1α	Hypoxia-Inducible Factor 1 Alpha
SERPINB2	Serpin Family B Member 2 (PAI-2)
AOD	Average Optical Density
IOD	Integrated Optical Density
APOA4	Apolipoprotein A4
CAR-T	Chimeric Antigen Receptor T Cell
CCL2	C-C Motif Chemokine Ligand 2
STAT1	Signal Transducer and Activator of Transcription 1
CXCL12	C-X-C Motif Chemokine Ligand 12
HLA	Human Leukocyte Antigen
JAK	Janus Kinase
ROS	Reactive Oxygen Species
iNOS	Inducible Nitric Oxide Synthase
cDC2	Conventional Type 2 Dendritic Cell
Bcl-2	B-cell Lymphoma 2
CCR4	C-C Chemokine Receptor Type 4
VSMC	Vascular Smooth Muscle Cell
C1QC	Complement C1q C Chain
LYVE1	Lymphatic Vessel Endothelial Hyaluronan Receptor 1
CD74	Cluster of Differentiation 74
IL-1Ra	Interleukin-1 Receptor Antagonist
VEGF-A	Vascular Endothelial Growth Factor A
PDGFRB	Platelet-Derived Growth Factor Receptor Beta
HIF3A	Hypoxia-Inducible Factor 3 Alpha
SQSTM1	Sequestosome 1
IP-10	Interferon-Inducible Protein 10
A2B	Adenosine A2B Receptor
COX-2	Cyclooxygenase-2
MIP-1α	Macrophage Inflammatory Protein-1 Alpha
IL-4	Interleukin-4
IL-12	Interleukin-12
IL-13	Interleukin-13
TCR	T Cell Receptor
SEER	Surveillance, Epidemiology, and End Results
SPR	Standardized Prevalence Ratio
SIR	Standardized Incidence Ratio
ICI	Immune Checkpoint Inhibitor
GAPDH	Glyceraldehyde-3-Phosphate Dehydrogenase
MDA5	Melanoma Differentiation-Associated Gene 5
IL-6R	Interleukin-6 Receptor
IL-6ST	Interleukin-6 Signal Transducer (gp130)
EAM	Experimental Autoimmune Myositis
SAMD	South Australian Myositis Database
nIMNM	Non-Immune-Mediated Necrotizing Myopathy
IMACS	International Myositis Assessment and Clinical Studies Group
CTCF	Corrected Total Cell Fluorescence
CD4	Cluster of Differentiation 4
CD8	Cluster of Differentiation 8
CD16	Cluster of Differentiation 16
CD19	Cluster of Differentiation 19
CD20	Cluster of Differentiation 20
CD56	Cluster of Differentiation 56
CD68	Cluster of Differentiation 68
CD138	Cluster of Differentiation 138
CD155	Cluster of Differentiation 155
CD163	Cluster of Differentiation 163
CD226	Cluster of Differentiation 226
M1	Classically Activated Macrophage
M2	Alternatively Activated Macrophage
C5	Complement Component 5
C1q	Complement Component 1q
MRI	Magnetic Resonance Imaging
